# Elucidating the invasion history of introduced bullfrogs in New Mexico using population genetic approaches

**DOI:** 10.7717/peerj.20491

**Published:** 2026-01-09

**Authors:** Celina M. Eberle, Daniele L.F. Wiley, Chris X. McDaniels, J. Tomasz Giermakowski, Lisa N. Barrow

**Affiliations:** Department of Biology, University of New Mexico, Albuquerque, NM, United States

**Keywords:** Bullfrog, *Rana catesbeiana*, Invasion genetics, New Mexico, Population genetics, Invasive species

## Abstract

The American Bullfrog, *Rana (Aquarana) catesbeiana*, is an extremely successful invader that has spread globally in the last century, impacting vulnerable ecosystems. In the western U.S., bullfrogs were intentionally introduced in the early 1900s, but little is known about their subsequent colonization into the state of New Mexico. We evaluated a single mitochondrial gene region, cytochrome b, using population genetic approaches to investigate the invasion history of bullfrogs across their introduced range. Specifically, our objectives were to (1) assess the level of genetic diversity and identify haplotypes within bullfrog populations in New Mexico, (2) compare the genetic diversity of native and invasive bullfrog populations, (3) infer the number of introductions into New Mexico, and (4) identify potential native source populations. Using haplotype and nucleotide diversity estimates, we found moderate genetic variation within New Mexico (*H*_*d*_ = 0.648, *π* = 0.0036) with higher diversity at sites with increased human activity. However, there was significantly lower genetic diversity in introduced populations compared to native populations, consistent with expectations of recent colonization. Based on haplotype diversity estimates and BLAST results, we found a total of eight haplotypes across New Mexico, of which six haplotypes were found across the native and other introduced ranges. Pairwise ΦST revealed minimal differentiation between New Mexico sites, consistent with introduction from a single source population. Lastly, the analysis of molecular variance (AMOVA) conveyed that bullfrogs from the Northwest (Northwest: 0%, *P* = 0.6411) and Southwest (Southwest: 0%, *P* = 0.5124) invasive regions showed no significant differences compared to New Mexico populations, suggesting either recent connectivity or similar origins. This study reinforces the importance of managing the movement of invasive species and demonstrates how evaluating the genetic composition of an invasive species can reveal key points of its invasion history.

## Introduction

The American Bullfrog, *Rana (Aquarana) catesbeiana*, is a successful invader that was initially introduced to non-native regions both intentionally, for recreational hunting as a food source (Brennan & Holycross, 2006), and unintentionally, through release from bullfrog aquaculture (Jennings & Hayes, 1985) and fish stockings (McAuliffe, 1978). Since then, American Bullfrogs (hereafter, bullfrogs) have expanded from their native range in the eastern United States of America (hereafter, U.S.) to the western U.S. and to more than 40 countries (Lowe et al., 2019). Listed in the IUCN’s Top 100 Most-Invasive Species, bullfrogs have been linked to species’ declines across the globe because of disease spread (Urbina et al., 2018), direct predation (Jancowski & Orchard, 2013), and competition for resources with native species (Snow & Witmer, 2010). This impact is especially prominent where native populations are already under high environmental stress due to a changing climate, including limited water availability and rising temperatures (Prăvălie, 2016).

The Southwestern U.S. is a climate-stressed region in which the interactions between invasive and native species have been relatively understudied (Archer & Predick, 2008; Dettinger, Udall & Georgakakos, 2015; Dueñas et al., 2018). Bullfrogs exacerbate environmental stressors by outcompeting native amphibians for limited aquatic habitat and preying on vulnerable populations (Hayes & Jennings, 1986; Hossack et al., 2023). For example, bullfrog establishment in the Southwestern U.S. has been directly linked to declines of the federally endangered Chiricahua Leopard Frog (*Rana chiricahuensis*) and Sonoran Tiger Salamander (*Ambystoma mavortium stebbinsi*) (United States Fish and Wildlife Service, 2002; United States Fish and Wildlife Service, 2007). In extreme environments such as the deserts of the American Southwest, where native species are already pushed to their physiological limits, the presence of a dominant invasive predator may further accelerate biodiversity loss (Hossack et al., 2023).

The invasion history of bullfrogs and their potential impacts on native ecosystems have been studied for over a decade, but little is known about the introduction of bullfrogs in the southwestern state of New Mexico. Bullfrogs are considered invasive across most of the state, though there has been considerable debate about how far west the native range spans. In New Mexico, bullfrogs are not native to the Rio Grande and the southwestern area of the state, but the northeastern edge of the state may be considered part of the native range (Degenhardt, Painter & Price, 1996). The first potential introduction of bullfrogs in New Mexico was described in a 1921 Game and Fish report (NMDGF, 1921), in which the State Game and Fish Commission instructed the Game Warden to secure bullfrogs from the Kansas State Fish Hatchery for stocking of the Rio Grande. Therefore, bullfrogs may have been introduced from the native range intentionally by government agencies for recreational hunting at least once. Records from annual reports between the years 1963–1968 and 1970–1971 (NMDGF, 1963; NMDGF, 1964; NMDGF, 1965; NMDGF, 1966; NMDGF, 1967; NMDGF, 1968; NMDGF, 1970; NMDGF, 1971) state that bullfrogs were acquired, kept on hand, and disposed of, which could indicate that more bullfrogs were introduced to New Mexico, but the release locations and source populations are unknown. Based on museum records, bullfrog populations far exceed the initial Rio Grande valley in New Mexico and have spread to disconnected aquatic habitats likely due to human-mediated dispersal, as in Kamath, Sepulveda & Layhee (2016) in Montana. To further investigate these unknowns, population genetics can be used to give first descriptions of the invasion history of bullfrogs into New Mexico and other introduced regions.

Population genetic analyses have successfully furthered our understanding of invasion biology across numerous taxa (*e.g.*, Baker & Stebbins, 1965; Lu et al., 2022; Thompson et al., 2024). For example, assessing haplotype frequency patterns across geographic space has proven helpful in identifying potential source populations and the number of introductions of zebra mussels into North America (May et al., 2006). Similar approaches have been applied to investigate global bullfrog introductions, demonstrating that source populations for bullfrogs in various European countries included midwestern and eastern native populations in the U.S. (Ficetola, Bonin & Miaud, 2008). Within the northwestern U.S., multiple introduction events from various native source regions were inferred and have likely contributed to the invasion success of bullfrogs in Montana (Kamath, Sepulveda & Layhee, 2016). Comparing the genetic diversity of invasive populations to their native counterparts provides insight into how genetic variation influences invasion success (Li et al., 2022). For example, genetic diversity can impact the success of invasive species by either limiting their ability to adapt to novel environmental stressors and putting them at a greater risk of extirpation when variation is low, or by enabling adaptive potential when variation is high (Kolbe et al., 2004).

Many invasive populations exhibit lower genetic variation compared to their native counterparts because of genetic bottlenecks associated with colonization (Estoup et al., 2016). Though this decreased variation should negatively impact their ability to colonize and adapt to new environments (Bouzat, 2010), many invasive populations can thrive. This scenario, known as the genetic paradox of invasions (Allendorf & Lundquist, 2003), has garnered sustained attention in recent years, with focused research into other mechanisms responsible for invasion success. For example, aspects such as phenotypic plasticity (Hagenblad et al., 2015) *via* transposable elements (Stapley, Santure & Dennis, 2015), host traits such as timing of reproduction (Urbina et al., 2020), or behavior related to recognizing predators (Garcia et al., 2012), have all been linked to invasion success despite low genetic diversity. One crucial factor in invasion success is the number of introductions, which can increase the genetic pool and promote successful establishment (Kolbe et al., 2004; Lavergne & Molofsky, 2007)*.* Bullfrogs demonstrate low genetic diversity in invasive populations with a variable number of introductions and source populations across North America (Funk et al., 2011; Kamath, Sepulveda & Layhee, 2016) and in other global studies (*e.g.*, China: Zhang et al., 2024), but they have been consistently successful in invading novel environments. These contrasting patterns highlight the complexity of identifying determinants of invasion success, emphasizing the need for further research to understand how genetic diversity, introduction history, and ecological context interact to shape establishment and spread.

The goal of this study was to use population genetics and phylogeographic approaches to uncover the invasion history of bullfrogs across their introduced range in the western U.S. with a focus on New Mexico. Specifically, we sequenced cytochrome b (cytb) in the mitochondrial genome of 77 individuals, sampled from multiple waterways across New Mexico. We then compiled and analyzed data from prior studies in other invasive and native regions and combined these with our new data to (1) assess the level of genetic diversity and identify haplotypes within bullfrog populations in New Mexico, (2) compare the genetic diversity of native and invasive bullfrog populations, (3) infer the number of introductions into New Mexico, and (4) identify potential native source populations. While we recognize that the inferential power of a single mitochondrial marker is limited, the use of this widely sequenced locus facilitated the synthesis of range-wide data and serves as an important first step for future invasive population studies of bullfrogs. By integrating genetic data from several previous studies into these analyses, this study provides insight into the mechanisms shaping bullfrog invasion history in the western U.S. and contributes to a broader understanding of how invasive species establish, spread, and persist in novel environments.

## Materials & Methods

### Sample collection

We obtained bullfrog tissue samples from museum loans and recent field collections. Field collections were completed under appropriate local and state permits: New Mexico Department of Game and Fish 3734, Rio Grande Nature Center 2023-010, and Bosque del Apache National Wildlife Refuge 2023-012. Samples archived at the Museum of Southwestern Biology (hereafter, MSB) at the University of New Mexico (UNM) were collected across the various river basins in northeastern and southwestern New Mexico between 2007–2022. Additional sampling was completed by us in the summer of 2023 within the Middle Rio Grande Basin and Lower Colorado River Basin. Specimen information is readily available for public access on the Arctos museum database (Table S1). Together the sampling consists of bullfrogs from Albuquerque (ABQ, *n* = 18), Socorro (SC, *n* = 3), Bosque del Apache (BDA, *n* = 4), Upper Gila River (UGR, *n* = 33), Cliff (CF, *n* = 4), Rodeo (RO, *n* = 14), and Mora River (MR, *n* = 1). We aimed to collect individuals from different ponds and along rivers approximately 500–1,000 m apart at sites to minimize collecting full sibling individuals. Individuals (various life stages: larval, juvenile, and adult) were caught by hand or dip net and transported to MSB where individuals were euthanized following approved University of New Mexico Institutional Animal Care and Use Committee Protocols (Protocols 20-201006-MC and 23-201375-MC). Specifically, we applied 20% benzocaine to the ventral side of the frog or tadpole until complete unresponsiveness was found using reflex tests, and then removed the heart and other tissues. Dissection tools were flame sterilized between frogs, and tissues were immediately flash frozen in liquid nitrogen and stored frozen until DNA extraction.

**Table 1:** Population genetic diversity metrics of introduced bullfrogs in New Mexico. Diversity metrics reported as haplotype diversity (*H*_*d*_) and nucleotide diversity (*π*). Population sample size (*n*) and number of haplotypes per population (*nH*_*d*_).

Population	*n*	*nH*_*d*_	*H*_*d*_	*π*
Albuquerque (ABQ)	18	6	0.83	0.009
Socorro (SC)	3	2	0.667	0.0014
Bosque del Apache (BDA)	4	2	0.5	0.0011
Upper Gila River (UGR)	33	2	0.435	0.0009
Cliff (CF)	4	3	0.833	0.0034
Rodeo (RO)	14	1	0	0
Mora River (MR)	1	1	NA	NA
All	77	17	0.648	0.0036

**Figure 1:**
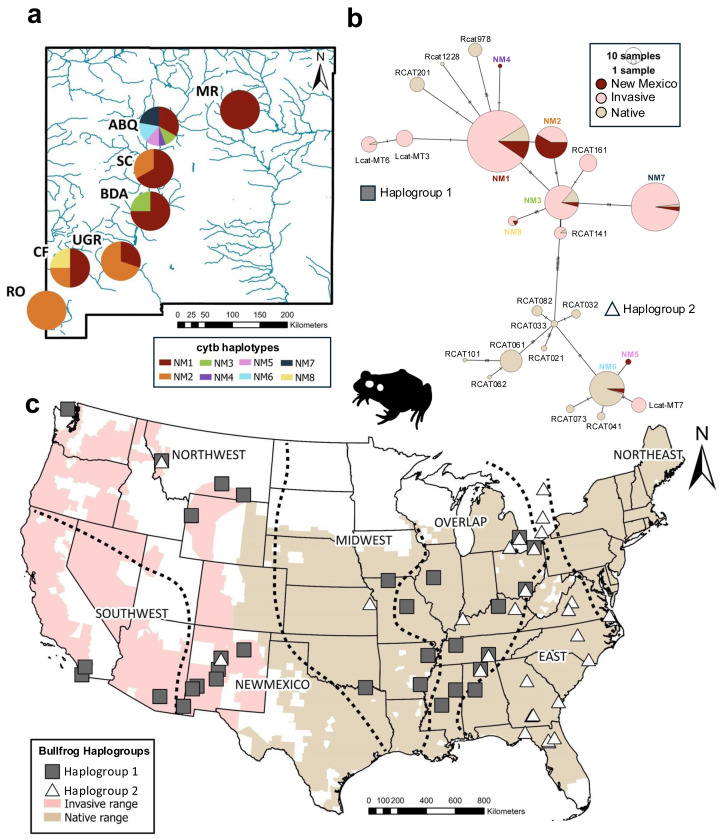
Haplotype and haplogroup distribution of bullfrogs sampled across the United States. (A) Cytochrome b haplotype proportions by sampling sites across New Mexico. Different haplotypes are represented by colors. (B) Haplotype network for cytochrome b haplotypes across the native and invasive range. Color indicates region where haplotype is found. Size of pie charts is proportional to haplotype freqency. Dashes on lines between haplotypes represents number of mutations. (C) Distributions of estimated bullfrog haplogroups according to phylogenetic analyses and haplotype network across invasive and native ranges. Silhouette of bullfrog was obtained from phylopic.org. U.S. County-level ranges were obtained from The International Union for Conservation of Nature’s Red List (IUCN, 2022).

### DNA extraction and sequencing

We extracted genetic DNA from liver, muscle, toe, and tail tissues using the E.Z.N.A. Tissue DNA Kit (Omega Bio-Tek, Norcross, GA, USA) following the manufacturer’s protocols. We used established polymerase chain reaction (PCR) protocols to amplify the mitochondrial gene, cytochrome b (cytb). Specifically, we amplified the 1,047-base pair (bp) gene region using the HERP328_cytb and HERP329_cytb forward and reverse primers (Yuan et al., 2016). This gene region has previously been used by other studies to investigate the invasion history of bullfrogs (Ficetola, Bonin & Miaud, 2008; Funk et al., 2011; Kamath, Sepulveda & Layhee, 2016) and to conduct phylogenetic analyses across the native bullfrog range (Austin, Lougheed & Boag, 2004).

We used 25 µL reaction volumes, each containing 30 ng of template DNA, one µL each of 10 mM forward and reverse primers, one µL of 25 mM MgCl2, one µL of 10 mM deoxynucleotide triphosphates (dNTPs), five µL of clear 5x GoTaq flexi buffer, 0.125 µL of GoTaq polymerase (Promega Corp., Madison, WI), and molecular-grade water. A negative control consisting of molecular grade water in place of template DNA was included with each batch of reactions. We ran reactions on a Bio-Rad T100 thermal cycler (Bio-Rad Laboratories, Hercules, CA) with the following thermal profile: an initial denaturation at 94 °C for 6 mins, 35 cycles of 94 °C for 20 secs, 52 °C for 30 secs, and 72 °C for 30 secs, and a final extension at 72 °C for 5 mins. The PCR products were visualized using a 1% agarose gel to confirm amplification of the gene region. Finally, PCR products were cleaned using Exo-SAP-IT (Bell, 2008) and sent for Sanger sequencing at Psomagen, Inc. (Rockville, MD).

### Haplotype comparisons

We edited and assembled forward and reverse sequences within each individual using Geneious version 2025.03 (Biomatters Ltd, Aukland, NZ). Raw forward and reverse sequences were trimmed to remove primers. Consensus sequences were then aligned using the Muscle alignment software implemented in Geneious (Kearse et al., 2012) with a reference bullfrog sequence from NCBI GenBank (AF205089.1). We collapsed the alignment of New Mexico sequences to unique haplotypes using the FaBox web tool (Villesen, 2007). We then used the standard nucleotide NCBI Basic Local Alignment Search Tool (BLAST; Altschul et al., 1990) to compare each New Mexico haplotype to publicly available sequences and identify previously sequenced individuals with identical haplotypes. Where possible, we determined the locations of those sequences from referenced literature or museum catalog numbers. We considered identical haplotypes based on both full-length sequences and shorter sequences (408 bp) available from previous studies.

### Population genetic diversity

We used the R package pegas (Paradis, 2010) to calculate genetic diversity metrics including haplotype diversity (*H*_*d*_) and nucleotide diversity (*π*), using the functions hap.div() and nuc.div(), respectively. We used both full-length and shorter sequences (408 bp) for these analyses. Diversity metrics were calculated using default settings for each New Mexico site except Mora River (MR), which was excluded because it consisted of a single individual. We compiled *H*_*d*_ and *π* metrics previously reported in tables and supplemental materials from the following studies to make comparisons between invasive and native populations: Ficetola, Bonin & Miaud, 2008; Funk et al., 2011; Kamath, Sepulveda & Layhee, 2016; LaFond et al., 2022. We computed the same genetic diversity metrics (*H*_*d*_ and *π*) for the native populations sampled in Austin, Lougheed & Boag (2004) using Arlequin 3.5 (Excoffier & Lischer, 2010) and modified supplemental files from Kamath, Sepulveda & Layhee (2016). These data are summarized by site in Table S2. We ran a Shapiro normality test to assess whether the diversity metrics followed a normal distribution. Neither index met that assumption; therefore, we decided to use Mann–Whitney U tests (Hart, 2001) *via* the wilcox.test() function to determine whether *H*_*d*_ or *π* differed between invasive and native populations. Effect sizes were then calculated with the rstatix package (Kassambara, 2023).

### Haplotype network and phylogenetic reconstruction

We assessed phylogenetic relationships of New Mexico haplotypes in comparison to native and other introduced haplotypes using sequences from GenBank. We selected one representative of each haplotype and included sequences that were longer than 600 bp (*n* = 35). The sequence alignment (full length = 949 bp) included both native (Austin, Lougheed & Boag, 2004; LaFond et al., 2022; MacGuigan et al., 2022) and invasive (Funk et al., 2011; Kamath, Sepulveda & Layhee, 2016; LaFond et al., 2022; Rodgers et al., 2023) bullfrog sequences.

We created a haplotype network in POPART v.1.7 using the Minimum Spanning Network (MSN) function for cytb sequences including one representative of each haplotype (haplotypes identical with New Mexico were combined) and included sequences longer than 600 bp (*n* = 27). We also used a maximum-likelihood (ML)-based approach to construct phylogenetic relationships among haplotypes using IQ-TREE version 2.3.6 (Nguyen et al., 2015). We used ModelFinder (Kalyaanamoorthy et al., 2017) to select an appropriate substitution model and UFBoot (Minh, Nguyen & von Haeseler, 2013) with 1,000 ultrafast bootstrap replicates to assess branch support. Nodes were considered well-supported with ML bootstrap support > 70% and we collapsed poorly supported nodes using TreeGraph 2 (Stöver & Müller, 2010). The outgroups used were *Rana heckscheri* and *Rana Clamitans* (GenBank Accessions AY083299 and AY083281).

### Evaluating introduction history

To identify the minimum number of introductions of bullfrogs to New Mexico, we estimated pairwise ΦST between each New Mexico population pair with significance evaluated based on 10,000 permutations. Analyses were conducted in Arlequin 3.5 (Excoffier & Lischer, 2010) using both full length and shorter sequences (408 bp). We applied a sequential Bonferroni correction (Holm, 1979) to correct for multiple pairwise tests (*n* = 15). Significant ΦST values indicate that two populations are genetically distinct in relation to haplogroups, which are interpreted as evidence of independent introductions (Ficetola, Bonin & Miaud, 2008; Funk et al., 2011). In contrast, nonsignificant ΦST values indicate a lack of genetic distinctiveness and therefore no support for independent introductions of those populations from separate sources (Waples & Gaggiotti, 2006).

We assessed possible source populations for New Mexico bullfrog populations using evidence from the following two approaches. First, we inferred potential source populations based on the geographic distribution of identical haplotypes within the native and invasive ranges outside of New Mexico. Second, we used an analysis of molecular variance (AMOVA) to yield statistical evidence of a potential source population (Excoffier, Smouse & Quattro, 1992). This analysis estimates the amount of genetic variation between groups of populations and requires subdivision of the potential source population range into meaningful biological areas. We grouped the native range into four meaningful areas (Midwest, Overlap, East, and Northeast) according to the nested clade analysis previously published by Austin, Lougheed & Boag (2004) and following the same framework as previous studies of other invasive regions (Ficetola, Bonin & Miaud, 2008; Funk et al., 2011; Kamath, Sepulveda & Layhee, 2016) (Fig. 1C). The Midwest region in our analysis corresponds to the West region in prior studies. We then divided the other invasive populations into two regions, Southwest and Northwest, based on their geography.

We ran the AMOVA analyses in Arlequin 3.5 using modified input files from Kamath, Sepulveda & Layhee (2016). We used all available sequences from Austin, Lougheed & Boag (2004), Funk et al. (2011), Kamath, Sepulveda & Layhee (2016), and LaFond et al. (2022) that were assigned to haplotype and site. The East region included 20 sites with 185 sequences, the Northeast included 15 sites with 68 sequences, the Overlap included 16 sites with 98 sequences, the Midwest included six sites with 33 sequences, the Northwest included 15 sites with 506 sequences, and the Southwest included seven sites with 130 sequences. Each of these regions was compared to New Mexico with six sites and 76 sequences (excluding Mora, which had a single sequence). Separate analyses were conducted to assess the amount of molecular variance attributed to differences between each region (Midwest, Overlap, East, Northeast, Southwest, or Northwest) and New Mexico. Significant among-group variation suggests that a region is unlikely to be a source population. We used 10,000 permutations to determine significance.

## Results

### Haplotype comparisons

**Figure 2:**
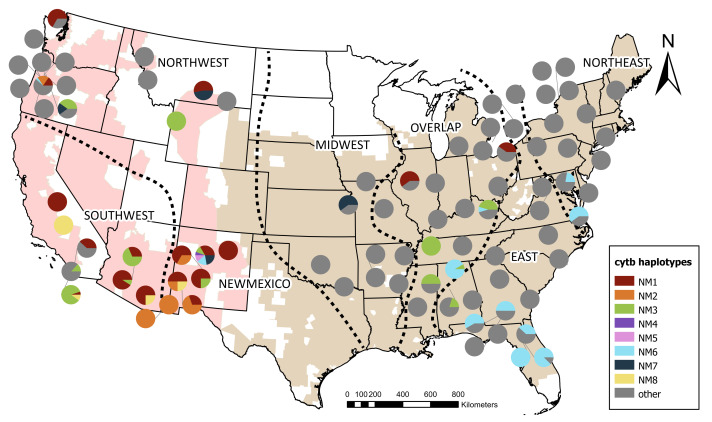
Distribution of cytochrome b haplotypes found in New Mexico bullfrogs across native and invasive ranges. Colors represent identical haplotypes to invasive populations found in New Mexico. Other haplotypes not identical to those in New Mexico are shown in grey. Both invasive and native ranges are shown on the map as shaded regions and were originally obtained from The International Union for Conservation of Nature’s Red List (IUCN, 2022) and adjusted according to new and existing museum records. The native range was divided into four meaningful biological areas, visualized by the dashed lines, based on the nested clade analysis published in Austin, Lougheed & Boag (2004). Additional sequences from Funk et al. (2010), Kamath, Sepulveda & Layhee, 2016, Lafond et al. (2022), MacGuigan et al. (2022), Rodgers et al. (2023) were assigned to these regions according to geography (Northeast, East, Overlap, Midwest). The invasive range was split into two regions (Northwest and Southwest) based on geographical boundaries.

We identified eight cytb haplotypes in bullfrogs across different sites in New Mexico (haplotypes NM1-NM8; Fig. 1A). Five haplotypes were restricted to a single site (NM4-NM7 in ABQ only; NM8 in CF only), while three haplotypes (NM1-NM3) were found at more than one site in NM. When we compared the full-length sequences of NM haplotypes to previous sequences, three were identical to haplotypes in the invasive populations in Montana and Wyoming (NM1 = MT1, NM3 = WY8, NM7 = MT2) and two haplotypes were identical to invasive bullfrogs sequenced in Arizona and California (NM2 = Rcat111, NM8 = Rcat162; Fig. 2). We also found two NM haplotypes that were identical to those from the native bullfrog range, one in the Overlap region in Mississippi (NM3 = RcatF = Rcat624) and one in the Northeast region in Maryland (NM6 = Rcat1415).

When the shorter, 408 bp sequences were compared, NM1 was identical to bullfrog sequences from the invasive range including California, Arizona, and Oregon; two from the native Overlap region in Ohio and Illinois; and one from Canada (Fig. 2). The short version of NM2 was identical to bullfrog sequences from Arizona and Oregon. The short NM3 sequence was identical to that of bullfrogs from the invasive range in Arizona, Oregon, and California, as well as within the Northeastern and Overlap native zones, including Mississippi, Tennessee, and Alabama. When only the short sequence is compared, NM4 was identical to NM1 and its matches in the invasive and native range. The short sequence of NM6 was identical to those in the Northeast, East, and Overlap native zones including states such as Alabama, Florida, Georgia, Virginia, and Maryland, and to sequences from the invasive range in the Northwest region in Oregon. The short version of NM7 was identical to sequences from the Midwest region of the native range, including Kansas, and from the invasive Northwest region, including Oregon and Montana. The short NM8 was identical to bullfrogs from the invasive range in California and Arizona. For both the full-length and short sequences, NM5 was not identical to any other haplotypes.

### Population genetic diversity

The Albuquerque and Cliff populations exhibited the highest genetic diversity among the New Mexico populations (ABQ: *H*_*d*_ = 0.830, *π* = 0.009; CF: *H*_*d*_ = 0.833, *π* = 0.0034; Table 1; Fig. 1A). We observed the lowest genetic diversity in the Upper Gila River population (UGR: *H*_*d*_ = 0.435, *n* = 0.0009) and in the Rodeo (RO) site, which had a single haplotype sequenced from 14 individuals. Overall, the mean haplotype diversity was moderately high for New Mexico, but the mean nucleotide diversity was low (Table 1).

**Figure 3:**
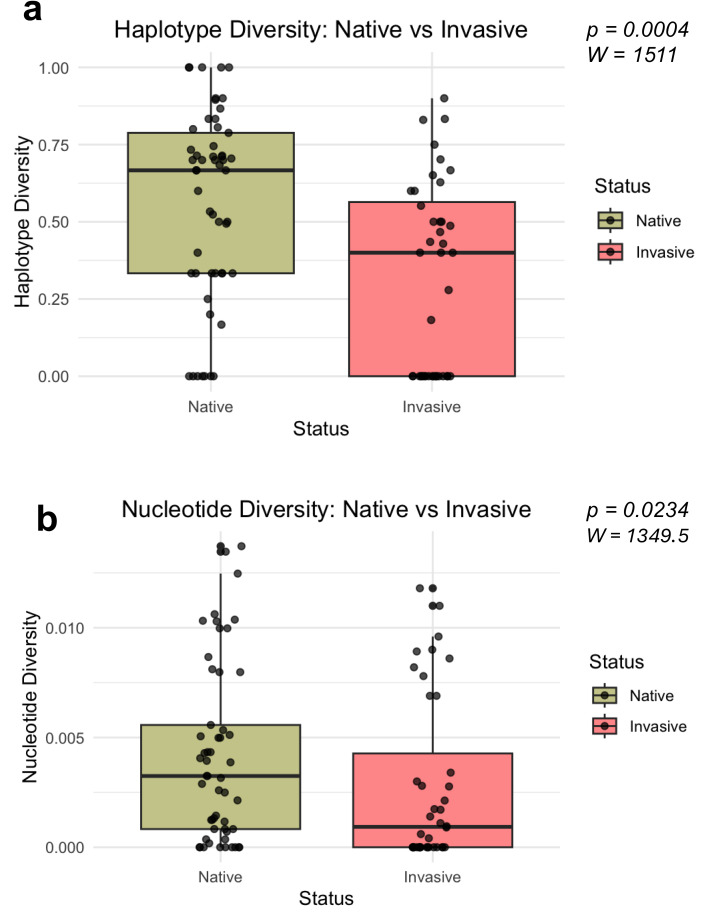
Distribution of genetic diversity metrics for native and invasive populations. Distribution of haplotype diversity (A) and nucleotide diversity (B) of native and invasive populations. Mann–Whitney U test statistics (W) and significance values (P) are provided for each comparison.

Mann–Whitney *U*-tests revealed significant differences in both haplotype diversity (*W* = 1511, *P* = 0.0004, *r* = 0.366) and nucleotide diversity (*W* = 1349.5, *P* = 0.0234, *r* = 0.236) among invasive and native bullfrog populations (Fig. 3). Both haplotype and nucleotide diversity were significantly lower in introduced populations compared to populations sampled from the native regions.

### Haplotype network and phylogenetic relationships

The haplotype network showed the cytb haplotypes of New Mexico bullfrog populations were grouped within the two haplogroups separated by 11 mutations (Fig. 1B). Most haplotypes (NM1, NM2, NM3, NM4, NM7, NM8) were within Haplogroup 1, while only two haplotypes (NM5, NM6) were within Haplogroup 2. Most haplotypes were only separated from other closely related haplotypes by 1–3 mutations. The best-fit substitution model for the phylogenetic analysis based on ModelFinder was K3Pu+F+I, which includes three substitution types, empirical base frequencies, and a proportion of invariable sites. Similar to the haplotype network, the maximum likelihood analysis inferred two cytb haplogroups with moderately high support (bootstrap value = 82, Haplogroup 1; bootstrap value = 80, Haplogroup 2, Fig. S1). These two haplogroups are broadly distributed across the native range (Fig. 1C) and have a large area of overlap. There is apparent clustering of New Mexico haplotypes within the network and phylogeny that could indicate a common source for NM1 and NM4, and for NM5 and NM6 (Fig. 1B; Fig. S1).

### Evaluating introduction history

Population genetic differentiation, measured as pairwise ΦST, between NM populations was low and not significant for most population pairs after sequential Bonferroni correction (Table 2). Two population pairs, ABQ-UGR and BDA-RO, were significantly differentiated after correction for multiple tests. However, based on chains of non-significant tests with other populations, the results suggest a minimum of one introduction to New Mexico.

**Table 2:** Pairwise ΦST and corresponding *P*-values between six introduced New Mexico populations. Pairwise ΦST values are found above the diagonal line and corresponding *P*-values are found below the diagonal line. Bolded text indicates significance after sequential Bonferroni correction for 15 total tests with a new significance level 0.0033.

	Albuquerque	Socorro	Bosque del Apache	Cliff	Upper Gila River	Rodeo
Albuquerque	—	0.057	0.072	0.053	0.217	0.249
Socorro	0.4079	—	0.043	−0.005	0.182	0.839
Bosque del Apache	0.3471	0.9999	—	0.047	0.452	0.901
Cliff	0.4605	0.9999	0.9999	—	0.282	0.645
Upper Gila River	0	0.5353	0.0137	0.0380	—	0.221
Rodeo	0.0040	0.0221	**0.0002**	0.0045	0.0372	—

The AMOVA revealed significant among-group variation between New Mexico bullfrog populations and three regions of the native range (Overlap: 39.8%*, P*= 0.0029, Northeast: 78.1%*, P <* 0.0001, East: 72.5%*, P <* 0.0001; Table 3), indicating these regions are unlikely source populations. Lower, marginally significant among-group variation was observed between New Mexico and the Midwest region within the native range (22%*, P*= 0.0503). The AMOVA also showed no among-group variation between New Mexico bullfrog populations and both regions in the invasive range (Northwest: 0%, *P* = 0.6411, Southwest: 0%, *P* = 0.5124).

**Table 3:** Summary of analysis of molecular variance (AMOVA). Results show the percentage of among-group variation between the introduced New Mexico population and the four native (*i.e.*, East, Northeast, Overlap, and Midwest) and two introduced (i.e., Northwest and Southwest) regions. Lower percentages indicate higher genetic similarity, while higher percentages indicate lower genetic similarity among groups. Bolded text indicates significance (*P* < 0.05).

Region compared to New Mexico	Percentage of among-group variation	*P-value*
East	72.45	**0.0001**
Northeast	78.14	**0.0001**
Overlap	39.77	**0.0029**
Midwest	22.04	0.0503
Northwest	0.0	0.6411
Southwest	0.0	0.5124

## Discussion

We investigated genetic differences of bullfrogs in New Mexico compared to other introduced and native populations to elucidate aspects of their invasion history. We found patterns consistent with a single introduction and recent expansion throughout the state of New Mexico, with little cytb differentiation among localities and most haplotypes matching those found in other native and invasive regions. Across New Mexico, genetic diversity was highest in localities with more human activity. Similar to previous studies of bullfrog invasion, we found low genetic diversity across introduced regions when compared to native populations, likely due to genetic bottlenecks associated with founder effects. While we were unable to identify the source population of New Mexico bullfrogs, in part because of limitations of the cytb marker, we found no significant among-group differences between New Mexico and other introduced western populations, which indicates a possible common origin or recent connectivity. Though records of bullfrog introduction into the western U.S. are disparate, our study provides new data and comparisons that suggest recent spread throughout the western U.S. and highlights the need for further research with higher-resolution genomic data.

### Genetic diversity in introduced and native populations

In this initial characterization of the genetic structure of New Mexico bullfrog populations, we found intriguing variation in genetic diversity among sites. Specifically, we found lower genetic diversity at more isolated sites (two well-sampled sites, RO and UGR, had only one and two haplotypes, respectively), while genetic diversity was higher in the site with increased human activity (ABQ had six haplotypes). These haplotypes occur within two different cytb groups found across the native bullfrog range, and ABQ is the only site we sampled that included haplotypes from both haplogroups. We note that the phylogenetic support for these groups was moderate (bootstrap values 80–82), which may be attributed to the relatively short gene fragment used. This pattern could be attributed to increased opportunities in urbanized areas for invasive species to move to different populations and increase genetic diversity (Santana Marques et al., 2020). Six haplotypes found across New Mexico were identical to haplotypes dispersed across the native and other introduced ranges, and one more was identical when the shorter 408bp sequence was compared. This similarity is consistent with a recent introduction, presumably within the last century (*e.g.*, NMDGF, 1921). The novel haplotypes we described could represent new mutations during that time or rare variants that would be revealed with more intense population sampling from other regions.

When comparing diversity metrics to populations from the native range, we found that invasive populations, including New Mexico, the Northwestern and Southwestern United States, and Europe, had significantly lower genetic diversity. This result corroborates previous studies that indicate successful bullfrog establishment is not constrained by lower genetic diversity (Ficetola, Bonin & Miaud, 2008; Funk et al., 2011; Bai et al., 2012; Kamath, Sepulveda & Layhee, 2016). Invasions are usually associated with genetic bottlenecks, which would be expected to limit the genetic diversity and adaptive potential of invasive populations (Ficetola, Bonin & Miaud, 2008; Estoup et al., 2016; Bors et al., 2019). Many studies have shown that multiple introductions from various regions could potentially introduce enough genetic diversity for an invasive species to thrive (Dlugosch & Parker, 2008; Winkler et al., 2019). For example, multiple introductions of Cuban brown anoles to Florida introduced higher genetic diversity compared to native counterparts that could be contributing to the success of invasive species (Kolbe et al., 2004). In bullfrogs, studies of other introduced populations in Montana (Kamath, Sepulveda & Layhee, 2016) and Europe (Ficetola, Bonin & Miaud, 2008) concluded there were multiple introductions, but with low genetic diversity.

In contrast, our data is consistent with one introduction of bullfrogs to New Mexico. Interestingly, New Mexico has several haplotypes that are identical to the native range, suggesting that a single invasive founding population could be from multiple sources that may have yielded enough genetic variation for the success and dispersal of bullfrogs across the state (Lavergne & Molofsky, 2007; Schrieber & Lachmuth, 2017). Many other factors could contribute to the success of invasive bullfrog populations despite low genetic variation. Their opportunistic predation habits, high mobility, and larger body size may confer advantages compared to native species (Snow & Witmer, 2010; Jancowski & Orchard, 2013). Furthermore, faster maturation time and presumed higher fecundity in introduced populations compared to native populations could provide a competitive advantage and maintain high population sizes (Urbina et al., 2020). Phenotypic plasticity may also be contributing to the success of bullfrogs in New Mexico enabling survival in new environmental conditions (Hagenblad et al., 2015). However, we caution that the inference of a single introduction is based on a single gene and ΦST estimates with large variances that may not be supported in future studies with more comprehensive genetic data, including additional genetic markers (Keller & Taylor, 2008; Sturk-Andreaggi et al., 2022). Sampling intensity has also been connected to the accuracy of ΦST estimates, therefore affecting the approximation of the number of introductions and gene flow (Dlugosch & Parker, 2008).

### Identifying potential sources and connectivity of introduced populations

Bullfrogs have been linked to various source populations and modes of introduction in previous studies. Many populations in China were attributed to frogs escaping bullfrog farms and food markets that were first imported from Cuba and Japan (Xuan, Yiming & McGarrity, 2010; Bai et al., 2012). In Montana and Wyoming, the native source population inferred was the Midwest, which may also be linked to bullfrog farming (Kamath, Sepulveda & Layhee, 2016), but other vectors such as the exotic pet trade and bait market for fishing, were also considered (Strecker, Campbell & Olden, 2011; Sepulveda et al., 2015). In Oregon, the native source population inferred was the Overlap region, which was attributed to a possible connection with bullfrog farms (Funk et al., 2011). In our study, we did not find conclusive evidence for a native source population, though the native group most similar to New Mexico, based on the AMOVA, was the Midwest, as in Kamath, Sepulveda & Layhee (2016). In addition, the AMOVA comparison found no significant differences between New Mexico and the invasive Northwest and Southwest regions, indicating a possible link between these populations. This finding could indicate a common source population for these introductions or a recent movement of populations from one invasive area to another.

While bullfrog farms may have contributed to introductions in the Northwest (Funk et al., 2011; Kamath, Sepulveda & Layhee, 2016), they are not a current threat for continued introduction because of their closures across North America (Helfrich, Neves & Parkhurst, 2009), nor are they likely to explain introductions to the Southwest. Historically, the Southwest did not have established bullfrog farms, but rather a record of intentional release and cultivation of bullfrogs for recreational hunting and fish stockings. In Arizona, bullfrogs were introduced for recreational hunting in the 1950s near Tucson and were stocked in rivers and lakes within a 100-mile radius (Brennan & Holycross, 2006). In California, bullfrogs were first introduced in 1912 for recreational hunting by a Louisiana dealer to the Amargosa River and were then moved to other aquatic environments in the Mojave Desert (Washington Department of Fish and Wildlife, 1995). There was also a separate introduction in California that involved a different population of bullfrogs from a San Francisco frog merchant in 1914, which was introduced into a nearby reservoir (Washington Department of Fish and Wildlife, 1995). In New Mexico, there was a similar history where the government intentionally released bullfrogs to the Rio Grande from a single population of bullfrogs from the Kansas State Fish Hatchery in 1921 (NMDGF, 1921).

The genetic similarities we identified among populations in New Mexico and the Southwest region, including California and Arizona, suggest a link between these populations. Arizona is geographically proximate to New Mexico and is connected through the Colorado River basin, but migration of bullfrogs over longer distances is less likely (Sepulveda & Layhee, 2015). Human-mediated dispersal is therefore a more probable explanation for the introduction and spread of bullfrogs across New Mexico. The identical haplotypes and lack of among-group variation between New Mexico and the Southwest region suggest another invasive population could have been a direct source. While our data is consistent with one single introduction to New Mexico, the current bullfrog range in the state far exceeds the Rio Grande, where bullfrogs were initially introduced, and the northeastern edge of the state, which may be part of the native range. Secondary spread after initial introduction is likely (Vander Zanden & Olden, 2008), and there is now greater concern to investigate secondary spread to improve management of New Mexico populations. Management strategies such as implementing laws to stop the import of invasive species into a region (Reed et al., 2023) and eradicating current populations (Hossack et al., 2023) have been successful in halting continued introduction and secondary spread in some states in the U.S. Current eradication efforts in New Mexico are focused on specific areas of overlap with vulnerable native species, but more concerted removal efforts and monitoring are critical to understanding the impacts of invasive bullfrogs across the state.

### Limitations and future directions

This study applied genetic approaches to provide a better understanding of the invasion history of an immensely successful invader. We provide key insights about the genetic composition of invasive bullfrog populations in the western U.S., including the first accounts of the invasion history in New Mexico. However, we recognize the limitations of using a single mtDNA gene region to uncover fine-scale dispersal patterns and gene flow (Hurst & Jiggins, 2005). Given the lower effective population size of mtDNA compared to nuclear DNA, this can affect inferences about genetic bottlenecks in population genetic studies (Ferreira & Rodriguez, 2024). Using additional genetic markers, including genome-wide loci, would improve resolution and enable the study of genetic adaptation (Sturk-Andreaggi et al., 2022). We also acknowledge that our sampling does not encompass all bullfrog populations, and varying sample sizes, especially low sample sizes in close geographic proximity, can affect measures of genetic diversity and haplotype estimates (Rosenberger et al., 2021). Increasing sample sizes and expanding sampling to include other populations would be helpful in future work. Our results provide insights into the connectivity of invasive populations and the importance of considering secondary spread to inform management strategies. Understanding the history of invasive species is critical in managing existing populations, and future work should continue monitoring population growth to avoid further declines of native species.

## Conclusions

The invasion history of bullfrogs across the globe has been widely studied, but little work has focused on the Southwestern U.S. Here, we provided the first genetic description of the bullfrog invasion history in this region and placed it in context with other invasive populations. Our results suggest successful bullfrog establishment may not be constrained by low genetic diversity. Specifically, we found that invasive populations consistently had lower genetic diversity than native populations. We also found limited genetic differentiation among sites in New Mexico, which is consistent with a single introduction event. This suggests that a single invasive founding population could have originated from multiple sources across the United States which contributed sufficient genetic diversity for widespread establishment. Given the widespread establishment of bullfrogs across New Mexico, future work should investigate the role of secondary spread. Additional genetic markers are needed to clarify a potential source population and help predict movement across the Southwest. Overall, our results provide insights into the connectivity of invasive bullfrog populations in New Mexico and highlight the importance of understanding the history of invasive species to managing existing populations.

## Supplemental Information

10.7717/peerj.20491/supp-1 Supplemental Information 1 Cytb Phylogenetic tree Maximum likelihood phylogeny estimated with cytochrome b sequence datasets shown as cladograms. Bootstrap values >70 are shown. All sequences are named after Genbank accession numbers and corresponding haplotype. The tree is rooted using Rana heckscheri and Rana clamitans as outgroups.

10.7717/peerj.20491/supp-2 Supplemental Information 2 Metadata of all New Mexico specimens This file contains Metadata of all New Mexico specimens used in this study from the Museum of Southwestern Biology. The file also contains links to the Arctos museum database where all information is readily available. The first sheet within the file contains important data for each specimen. Definitions of column headers are included on the second sheet.

10.7717/peerj.20491/supp-3 Supplemental Information 3 Cytb sequence data The data for sequences per site used in the study including diversity indices, sample sizes and geographical data. This also exhibits the number of populations compared, the AMOVA region, and which study each population was from.

## Additional Information and Declarations

### Field Study Permissions

The following information was supplied relating to field study approvals (i.e., approving body and any reference numbers): Specimen collection was approved by the New Mexico Department of Game and Fish, Rio Grande Nature Center, and Bosque del Apache National Wildlife Refuge.

### DNA Deposition

The following information was supplied regarding the deposition of DNA sequences: The New Mexico cytb bullfrog sequences are available at GenBank: PV261955 to PV262031.

### Data Availability

The following information was supplied regarding data availability: All supporting data, R codes and input files, are available at Figshare: Eberle, Celina (2025). Using genetic approaches to uncover the invasion history of bullfrogs in the Southwest. figshare. Dataset. https://doi.org/10.6084/m9.figshare.29285741.v2.

## References

[ref-1] Allendorf FW, Lundquist LL (2003). Introduction: population biology, evolution, and control of invasive species. Conservation Biology.

[ref-2] Altschul SF, Gish W, Miller W, Myers EW, Lipman DJ (1990). Basic local alignment search tool. Journal of Molecular Biology.

[ref-3] Archer SR, Predick KI (2008). Climate change and ecosystems of the Southwestern United States. Rangelands.

[ref-4] Austin JD, Lougheed SC, Boag PT (2004). Discordant temporal and geographic patterns in maternal lineages of eastern north American frogs, *Rana catesbeiana* (*Ranidae*) and *Pseudacris crucifer* (*Hylidae*). Molecular Phylogenetics and Evolution.

[ref-5] Bai C, Ke Z, Consuegra S, Liu X, Li Y (2012). The role of founder effects on the genetic structure of the invasive bullfrog (*Lithobates catesbeianaus*) in China. Biological Invasions.

[ref-6] Baker HG, Stebbins GL (1965). The genetics of colonizing species.

[ref-7] Bell JR (2008). A simple way to treat PCR products prior to sequencing using ExoSAP-IT^®^. BioTechniques.

[ref-8] Bors EK, Herrera S, Morris Jr JA, Shank TM (2019). Population genomics of rapidly invading lionfish in the Caribbean reveals signals of range expansion in the absence of spatial population structure. Ecology and Evolution.

[ref-9] Bouzat JL (2010). Conservation genetics of population bottlenecks: the role of chance, selection, and history. Conservation Genetics.

[ref-10] Brennan TC, Holycross AT (2006). A field guide to amphibians and reptiles in Arizona.

[ref-11] Degenhardt WG, Painter CW, Price AH (1996). Amphibians and reptiles of New Mexico.

[ref-12] Dettinger M, Udall B, Georgakakos A (2015). Western water and climate change. Ecological Applications.

[ref-13] Dlugosch KM, Parker IM (2008). Founding events in species invasions: genetic variation, adaptive evolution, and the role of multiple introductions. Molecular Ecology.

[ref-14] Dueñas M-A, Ruffhead HJ, Wakefield NH, Roberts PD, Hemming DJ, Diaz-Soltero H (2018). The role played by invasive species in interactions with endangered and threatened species in the United States: a systematic review. Biodiversity and Conservation.

[ref-15] Estoup A, Ravigné V, Hufbauer R, Vitalis R, Gautier M, Facon B (2016). Is there a genetic paradox of biological invasion?. Annual Review of Ecology, Evolution, and Systematics.

[ref-16] Excoffier L, Lischer HEL (2010). Arlequin suite ver 3.5: a new series of programs to perform population genetics analyses under Linux and Windows. Molecular Ecology Resources.

[ref-17] Excoffier L, Smouse PE, Quattro JM (1992). Analysis of molecular variance inferred from metric distances among DNA haplotypes: application to human mitochondrial DNA restriction data. Genetics.

[ref-18] Ferreira T, Rodriguez S (2024). Mitochondrial DNA: inherent complexities relevant to genetic analyses. Gene.

[ref-19] Ficetola GF, Bonin A, Miaud C (2008). Population genetics reveals origin and number of founders in a biological invasion. Molecular Ecology.

[ref-20] Funk WC, Garcia TS, Cortina GA, Hill RH (2011). Population genetics of introduced bullfrogs, Rana (*Lithobates*) *catesbeianus*, in the Willamette Valley, Oregon, USA. Biological Invasions.

[ref-21] Garcia TS, Thurman LL, Rowe JC, Selego SM (2012). Antipredator behavior of American Bullfrogs (*Lithobates catesbeianus*) in a novel environment. Ethology.

[ref-22] Hagenblad J, Hülskötter J, Acharya KP, Brunet J, Chabrerie O, Cousins SAO, Dar PA, Diekmann M, De Frenne P, Hermy M, Jamoneau A, Kolb A, Lemke I, Plue J, Reshi ZA, Graae BJ (2015). Low genetic diversity despite multiple introductions of the invasive plant species *Impatiens glandulifera* in Europe. BMC Genetics.

[ref-23] Hart A (2001). Mann–Whitney test is not just a test of medians: differences in spread can be important. BMJ: British Medical Journal.

[ref-24] Hayes MP, Jennings MR (1986). Decline of ranid frog species in Western North America: are Bullfrogs (*Rana catesbeiana*) responsible?. Journal of Herpetology.

[ref-25] Helfrich LA, Neves RJ, Parkhurst JA (2009). Commercial frog farming (Nos. 420–255).

[ref-26] Holm S (1979). A simple sequentially rejective multiple test procedure. Scandinavian Journal of Statistics.

[ref-27] Hossack BR, Hall D, Crawford CL, Goldberg CS, Muths E, Sigafus BH, Chambert T (2023). Successful eradication of invasive American bullfrogs leads to coextirpation of emerging pathogens. Conservation Letters.

[ref-28] Hurst GDD, Jiggins FM (2005). Problems with mitochondrial DNA as a marker in population, phylogeographic and phylogenetic studies: the effects of inherited symbionts. Proceedings of the Royal Society B: Biological Sciences.

[ref-29] IUCN (2022). The IUCN red list of threatened species. Version 2022. https://www.iucnredlist.org.

[ref-30] Jancowski K, Orchard S (2013). Stomach contents from invasive American bullfrogs *Rana catesbeiana* (= *Lithobates catesbeianus*) on southern Vancouver Island, British Columbia, Canada. NeoBiota.

[ref-31] Jennings MR, Hayes MP (1985). Pre-1900 overharvest of California red-legged frogs (*Rana aurora draytonii*): the inducement for Bullfrog (*Rana catesbeiana)* introduction. Herpetologica.

[ref-32] Kalyaanamoorthy S, Minh BQ, Wong TKF, Von Haeseler A, Jermiin LS (2017). ModelFinder: fast model selection for accurate phylogenetic estimates. Nature Methods.

[ref-33] Kamath PL, Sepulveda AJ, Layhee M (2016). Genetic reconstruction of a bullfrog invasion to elucidate vectors of introduction and secondary spread. Ecology and Evolution.

[ref-34] Kassambara A (2023). https://CRAN.R-project.org/package=rstatix.

[ref-35] Kearse M, Moir R, Wilson A, Stones-Havas S, Cheung M, Sturrock S, Buxton S, Cooper A, Markowitz S, Duran C, Thierer T, Ashton B, Meintjes P, Drummond A (2012). Geneious basic: an integrated and extendable desktop software platform for the organization and analysis of sequence data. Bioinformatics.

[ref-36] Keller SR, Taylor DR (2008). History, chance and adaptation during biological invasion: separating stochastic phenotypic evolution from response to selection. Ecology Letters.

[ref-37] Kolbe JJ, Glor RE, Rodríguez Schettino L, Lara AC, Larson A, Losos JB (2004). Genetic variation increases during biological invasion by a Cuban lizard. Nature.

[ref-38] LaFond J, Martin KR, Dahn H, Richmond JQ, Murphy RW, Rollinson N, Savage AE (2022). Invasive Bullfrogs maintain MHC polymorphism including alleles associated with chytrid fungal infection. Integrative and Comparative Biology.

[ref-39] Lavergne S, Molofsky J (2007). Increased genetic variation and evolutionary potential drive the success of an invasive grass. Proceedings of the National Academy of Sciences of the United States of America.

[ref-40] Li F, Liu X, Zhu J, Li J, Gao K, Zhao C (2022). The role of genetic factors in the differential invasion success of two spartina species in China. Frontiers in Plant Science.

[ref-41] Lowe S, Browne M, Boudjelas S, De Poorter M (2019). 100 of the world’s worst invasive alien species: a selection from the global invasive species database. Encyclopedia of biological invasions.

[ref-42] Lu S, Luo X, Han L, Yang J, Jin J, Yang J (2022). Genetic patterns reveal differences between the invasion processes of common ragweed in urban and non-urban ecosystems. Global Ecology and Conservation.

[ref-43] MacGuigan DJ, Mount GG, Watkins-Colwell GJ, Near TJ, Lambert MR (2022). Genomic data clarify aquarana systematics and reveal isolation-by-distance dominates phylogeography of the wide-ranging frog *Rana clamitans*. Ichthyology & Herpetology.

[ref-44] May GE, Gelembiuk GW, Panov VE, Orlova MI, Lee CE (2006). Molecular ecology of zebra mussel invasions. Molecular Ecology.

[ref-45] McAuliffe JR (1978). Biological survey and management of sport-hunted Bullfrog populations in Nebraska.

[ref-46] Minh BQ, Nguyen MAT, Von Haeseler A (2013). Ultrafast approximation for phylogenetic bootstrap. Molecular Biology and Evolution.

[ref-47] Nguyen L-T, Schmidt HA, Von Haeseler A, Minh BQ (2015). IQ-TREE: a fast and effective stochastic algorithm for estimating maximum-likelihood phylogenies. Molecular Biology and Evolution.

[ref-48] NMDGF (1921). Report of the game and fish warden of New Mexico.

[ref-49] NMDGF (1963). 1963 Report of the game and fish warden of New Mexico.

[ref-50] NMDGF (1964). 1964 Report of the game and fish warden of New Mexico.

[ref-51] NMDGF (1965). 1965 Report of the game and fish warden of New Mexico.

[ref-52] NMDGF (1966). 1966 Report of the game and fish warden of New Mexico.

[ref-53] NMDGF (1967). 1967 Report of the game and fish warden of New Mexico.

[ref-54] NMDGF (1968). 1968 Report of the game and fish warden of New Mexico.

[ref-55] NMDGF (1970). 1970 Report of the game and fish warden of New Mexico.

[ref-56] NMDGF (1971). 1971 Report of the game and fish warden of New Mexico.

[ref-57] Paradis E (2010). pegas: an R package for population genetics with an integrated-modular approach. Bioinformatics.

[ref-58] Prăvălie R (2016). Drylands extent and environmental issues. A global approach. Earth-Science Reviews.

[ref-59] Reed EMX, Cathey S, Braswell C, Agarwal P, Barney JN, Brown BL, Heminger A, Kianmehr A, Salom S, Schenk T, Sharma G, Haak DC (2023). The state of play in invasive species policy: insights from invasive species laws and regulations in 21 US states. BioScience.

[ref-60] Rodgers TW, Lovich RE, Walton JA, Prince DJ, Gonzalez BR, Shaffer HB, Mock KE (2023). Multiplex qPCR assays for detection of 2 imperiled anuran species, Anaxyrus californicus and *Spea hammondii*, from environmental DNA. Freshwater Science.

[ref-61] Rosenberger K, Schumacher E, Brown A, Hoban S (2021). Proportional sampling strategy often captures more genetic diversity when population sizes vary. Biological Conservation.

[ref-62] Santana Marques P, Resende Manna L, Clara Frauendorf T, Zandonà E, Mazzoni R, El-Sabaawi R (2020). Urbanization can increase the invasive potential of alien species. Journal of Animal Ecology.

[ref-63] Schrieber K, Lachmuth S (2017). The genetic paradox of invasions revisited: the potential role of inbreeding × environment interactions in invasion success. Biological Reviews.

[ref-64] Sepulveda AJ, Layhee M (2015). Description of fall and winter movements of the introduced American Bullfrog (*Lithobates catesbeianus*) in a Montana, USA, Pond. Herpetological Conservation and Biology.

[ref-65] Sepulveda A, Layhee M, Stagliano D, Chaffin J, Begley A, Maxell B (2015). Invasion of American bullfrogs along the Yellowstone River. Aquatic Invasions.

[ref-66] Snow NP, Witmer G (2010). American bullfrogs as invasive species: a review of the introduction, subsequent problems, management options, and future directions.

[ref-67] Stapley J, Santure AW, Dennis SR (2015). Transposable elements as agents of rapid adaptation may explain the genetic paradox of invasive species. Molecular Ecology.

[ref-68] Stöver BC, Müller KF (2010). TreeGraph 2: combining and visualizing evidence from different phylogenetic analyses. BMC Bioinformatics.

[ref-69] Strecker AL, Campbell PM, Olden JD (2011). The aquarium trade as an invasion pathway in the Pacific Northwest. Fisheries.

[ref-70] Sturk-Andreaggi K, Ring JD, Ameur A, Gyllensten U, Bodner M, Parson W, Marshall C, Allen M (2022). The value of whole-genome sequencing for mitochondrial DNA population studies: strategies and criteria for extracting high-quality mitogenome haplotypes. International Journal of Molecular Sciences.

[ref-71] Thompson B, Atsawawaranunt K, Nehmens MC, Pearman WS, Perkins EO, Pipek P, Rollins LA, Tan HZ, Whibley A, Santure AW, Stuart KC (2024). Population genetics and invasion history of the European Starling across Aotearoa New Zealand. Molecular Ecology.

[ref-72] Urbina J, Bredeweg EM, Cousins C, Blaustein AR, Garcia TS (2020). Reproductive characteristics of American bullfrogs (*Lithobates catesbeianus)* in their invasive range of the Pacific Northwest, USA. Scientific Reports.

[ref-73] Urbina J, Bredeweg EM, Garcia TS, Blaustein AR (2018). Host-pathogen dynamics among the invasive American bullfrog (*Lithobates catesbeianus*) and chytrid fungus (*Batrachochytrium dendrobatidis*). Hydrobiologia.

[ref-74] United States Fish and Wildlife Service (2002). Sonora tiger salamander recovery plan.

[ref-75] United States Fish and Wildlife Service (2007). Chiricahua Leopard Frog (*Rana chiricahuensis)* final recovery plan.

[ref-76] Vander Zanden MJ, Olden JD (2008). A management framework for preventing the secondary spread of aquatic invasive species. Canadian Journal of Fisheries and Aquatic Sciences.

[ref-77] Villesen P (2007). FaBox: an online toolbox for fasta sequences. Molecular Ecology Notes.

[ref-78] Waples RS, Gaggiotti O (2006). Invited review: what is a population? An empirical evaluation of some genetic methods for identifying the number of gene pools and their degree of connectivity. Molecular Ecology.

[ref-79] Washington Department of Fish and Wildlife (1995). Report for United States Fish and Wildlife Service, Washington D.C..

[ref-80] Winkler DE, Chapin KJ, François O, Garmon JD, Gaut BS, Huxman TE (2019). Multiple introductions and population structure during the rapid expansion of the invasive Sahara mustard (*Brassica tournefortii)*. Ecology and Evolution.

[ref-81] Xuan L, Yiming L, McGarrity M (2010). Geographical variation in body size and sexual size dimorphism of introduced American bullfrogs in Southwestern China. Biological Invasions.

[ref-82] Yuan Z-Y, Zhou W-W, Chen X, Poyarkov NA, Chen H-M, Jang-Liaw N-H, Chou W-H, Matzke NJ, Iizuka K, Min M-S, Kuzmin SL, Zhang Y-P, Cannatella DC, Hillis DM, Che J (2016). Spatiotemporal diversification of the true frogs (Genus *Rana*): a historical framework for a widely studied group of model organisms. Systematic Biology.

[ref-83] Zhang J, Xu C, Wang S, Wang S, Li Y (2024). Variations in genetic diversity of invasive species *Lithobates catesbeianus* in China. Animals.

