# Supplementary figures and images for: Elucidating the invasion history of introduced bullfrogs in New Mexico using population genetic approaches
Source: PeerJ. 2026 Jan 9;14:e20491. doi: 10.7717/peerj.20491 (PMC12794634; doi:10.7717/peerj.20491)

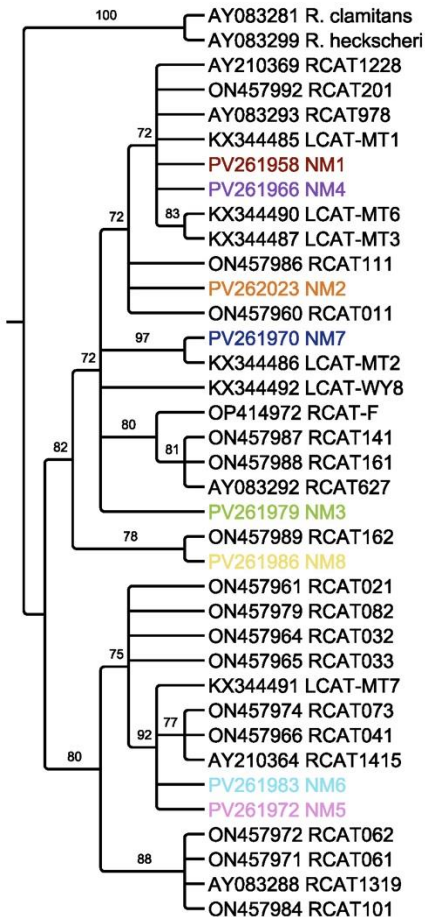

Haplogroup 1

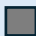

Haplogroup 2

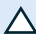

### cytb haplotypes

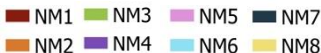

Supplement: Supplemental Information 1 — Maximum likelihood phylogeny estimated with cytochrome b sequence datasets shown as cladograms. Bootstrap values >70 are shown. All sequences are named after Genbank accession numbers and corresponding haplotype. The tree is rooted using Rana heckscheri and Rana clamitans as outgroups. [file peerj-14-20491-s001.pdf]
